# Interventional radiology in the management of portal hypertension

**DOI:** 10.4103/0971-3026.41840

**Published:** 2008-08

**Authors:** Sundeep J Punamiya

**Affiliations:** Department of Radiology, Tan Tock Seng Hospital, 11 Jalan Tan Tock Seng, 308433, Singapore

**Keywords:** Interventional radiology, portal hypertension

## Abstract

From being a mere (though important) diagnostic tool, radiology has evolved to become an integral part of therapy in portal hypertension today. Various procedures are currently available, the choice depending on the etiology and location of disease, the pathoanatomy, and the symptomatology. The main aim of any procedure is to reduce the portal pressure by either direct or indirect methods. This can be achieved with transjugular intrahepatic portosystemic shunt (TIPS), recanalization of the hepatic vein outflow, recanalization of the portal vein and its tributaries, recanalization of dysfunctional portosystemic shunts, partial splenic embolization, and embolization of arterioportal shunts. When any of these procedures cannot be performed due to anatomical or physiological reasons, the symptoms can often be controlled effectively with embolization of varices or balloon-occluded retrograde transvenous obliteration of varices (BRTO). This article briefly describes the procedures, their results, and their current status in the treatment of portal hypertension.

The role of the radiologist in the management of portal hypertension (PHT) has undergone a significant metamorphosis over the last few decades. Initially, it was limited to determining the presence and cause of PHT, using angiographic techniques such as percutaneous splenoportography, transhepatic portography, and arterioportography. For many years these procedures were conducted frequently for planning surgical treatment; later, they were replaced by safer and equally reliable modalities such as USG, CT scan and CT angiography (CTA) and MRI and MRI angiography (MRA).

Progressively, the focus changed from diagnostic to therapeutic procedures. Interventions involving the portal venous system were introduced in the 1970s, beginning with transhepatic embolization for control of gastric and esophageal variceal bleeding. The subsequent decade saw an expansion in the variety of therapeutic interventions, with procedures such as transjugular intrahepatic portosystemic shunt, portal vein recanalization, balloon-occluded retrograde transvenous obliteration of varices, hepatic venous outflow angioplasty, and revision of surgical shunts being rapidly introduced one after the other. Since then radiological interventions have become established methods in the treatment of PHT.

## Interventions in PHT

The primary goal in treating portal hypertension is reduction in the portal venous pressure itself. This should mitigate the complications of portal hypertension such as bleeding from varices and congestive gastroenteropathy, accumulation of ascites and hydrothorax, or the hepatorenal syndrome, etc. When it is not possible to achieve this primary goal, various procedures can be offered to palliate or control the symptoms related to portal hypertension. The various portal vein interventions can be broadly categorized as:

1. Interventions that reduce portal blood pressure:
  - Transjugular intrahepatic portosystemic shunts (TIPS)
  - Recanalization of hepatic venous outflow
  - Recanalization of the occluded portal vein and its tributaries
  - Embolization of arterioportal fistula
  - Partial splenic embolization
  - Revision of occluded surgical or radiological portosystemic shunts
2. Interventions to palliate symptoms related to portal hypertension (without altering the portal blood pressure):
  - Percutaneous transhepatic variceal embolization
  - Balloon retrograde obliteration of gastric varices (BRTO)
  - Percutaneous peritoneovenous shunt

### Transjugular intrahepatic portosystemic shunt (TIPS)

**Figure 1 (A, B):**
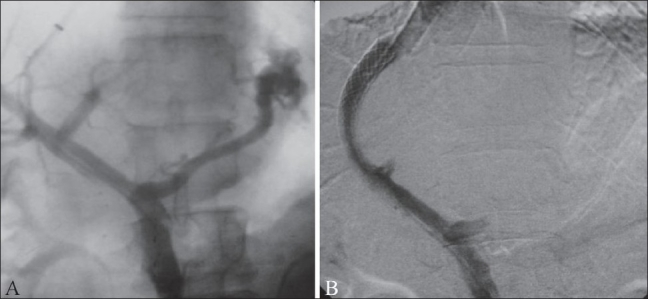
TIPS performed in a patient with uncontrolled varicealbleed. Portal venogram (A) obtained after puncture of the portal vein shows retrograde filling of the left gastric vein and feeding of a large junctional varix. The portosystemic gradient was recorded at 21 mm Hg. The post-TIPS venogram (B) shows good flow across the TIPS. Adequate decompression is evident from the non-fi lling of the left gastric vein and varices and reduction of the portosystemic gradient to 4 mm Hg

TIPS is a portosystemic shunt created within the liver parenchyma between the hepatic vein and the portal vein. The procedure involves many steps: (1) puncture of the jugular vein, (2) cannulation of the hepatic vein, (3) passage of a long needle from the hepatic vein, through the liver parenchyma and into the portal vein, (4) dilatation, with an angioplasty balloon, of the parenchymal tract created by the needle, and (5) stent deployment to ensure patency of the tract. Usually an 8-10 mm diameter stent is chosen to adequately decompress the hypertensive portal circulation to achieve a final portosystemic gradient of less than 12 mm Hg [Figure 1].

TIPS was conceived as early as 1969, when Rösch reported this method in a series of dog experiments using Teflon tubes as stents.[1] It has since then matured from an experimental procedure into an established technique and has replaced surgical shunts in most centers where it is available. It has proved to be effective in treating various complications of PHT [Table 1].[2]

**Table 1:** Indications of transjugular intrahepatic portosystemic shunt

1.	Acute variceal bleeding unresponsive to medical and endoscopic therapy
2.	Recurrent variceal bleeding unresponsive to medical and endoscopic therapy
3.	Ectopic variceal bleeding (e.g., bleeding from duodenal varices, rectal varices, stomal varices, and caput medusae)
4.	Nonvariceal bleeding secondary to hypertensive gastropathy/enteropathy
5.	Ascites resistant or intolerant to optimal medical therapy
6.	Hepatic hydrothorax resistant or intolerant to optimal medical therapy
7.	Budd-Chiari syndrome
8.	Hepatorenal syndrome
9.	Hepatopulmonary syndrome

TIPS is contraindicated in patients with congestive heart failure, severe pulmonary hypertension, severe tricuspid regurgitation, hepatic failure, preexisting encephalopathy, unrelieved biliary obstruction, multiple hepatic cysts, and uncontrolled systemic infection. Relative contraindications include the presence of large liver tumors, hepatic vein thrombosis, portal vein thrombosis, thrombocytopenia (< 20,000/cm^3^) and severe coagulopathy (INR >5).[2]

TIPS is successful in more than 95% of patients and is generally safe, with a procedural mortality of < 1%. The 1-month mortality, however, is variable and can be as high as 40%, in patients with limited hepatic reserve. This poor outcome can be predicted using various prognostic indicators such as serum bilirubin (>3 mg/dl), Child-Pugh score (>12), modified MELD score (>25), APACHE II score (>18), or Emory risk score (>3).[3]

TIPS gives excellent short-term results by controlling bleeding in >90% cases and controlling ascites and hydrothorax in >70% cases; these results are much better than those obtained by more traditional methods of therapy, i.e., endoscopic therapy (ET) and repeated paracentesis [Tables 2 and 3].[4–21]

**Table 2:** Results of randomized trials of transjugular intrahepatic portosystemic shunt *vs* endoscopic therapy

Author	No of patients		Type of ET	Rebleed rate		Mortality rate		Encephalopathy	
					
	TIPS	ET		TIPS	ET	TIPS	ET	TIPS	ET
GEAIH*	32	33	Sclerotherapy	40.6	60.6	50.0	42.4	NA	NA
Cabrera	31	32	Sclerotherapy	22.6	50.0	19.3	15.6	3.2	12.5
Rossle	61	65	Sclerotherapy/ligation + propranolol	14.8	44.6	13.1	12.3	29.5	13.8
Sanyal	41	39	Sclerotherapy	21.9	20.5	29.3	17.9	29.3	12.8
Cello	24	25	Sclerotherapy	12.5	48	33.3	32	50	44
Sauer	42	41	Sclerotherapy + propanolol	14.3	51.2	28.6	26.8	33.3	7.3
Jalan	31	27	Band ligation	9.7	55.6	41.9	37	16.1	11.1
Merli	38	43	Sclerotherapy	18.4	39.5	23.7	18.6	55.3	23.2
Sauer	43	42	Band ligation	16.3	42.9	25.6	28.6	37.2	21.4
Garcia-Villareal	22	24	Sclerotherapy	9.0	50.0	13.6	33.3	22.7	25.0
Pomier-Layrargues	41	39	Band ligation	19.5	56.4	41.5	41	36.6	41
Narahara	38	40	Sclerotherapy	18.4	32.5	28.9	17.5	34.2	15
Gulberg	28	26	Band ligation	25.0	26.9	14.3	15.4	7.1	3.8
Mean				27.3	44.5	29.0	26.0	29.5	19.2

* Groupe d'Etude des Anastomoses Intra-Hepatiques TIPS - Transjugular intrahepatic portosystemic shunt, ET - Endoscopic therapy

**Table 3:** Results of randomized trials comparing transjugular intrahepatic portosystemic shunt with repeated large-volume paracentesis

Author	No of patients		Control of ascites (at 4 months)		Survival (at 12 months)		Encephalopathy	
				
	TIPS	LVP	TIPS	LVP	TIPS	LVP	TIPS	LVP
Rossle	29	31	70.0	22.6	69.0	51.6	58.6	48.4
Gines	35	35	65.7	8.6	40.0	34.3	77.1	65.7
Sanyal	52	57	59.6	15.8	71.2	71.9	42.3	22.8
Salerno	33	33	84.8	63.6	75.6	51.5	60.6	39.4
Mean			70.0	27.6	64.0	52.3	59.7	44.1

TIPS - Transjugular intrahepatic portosystemic shunt, LVP - Large-volume paracentesis

**Figure 2:**
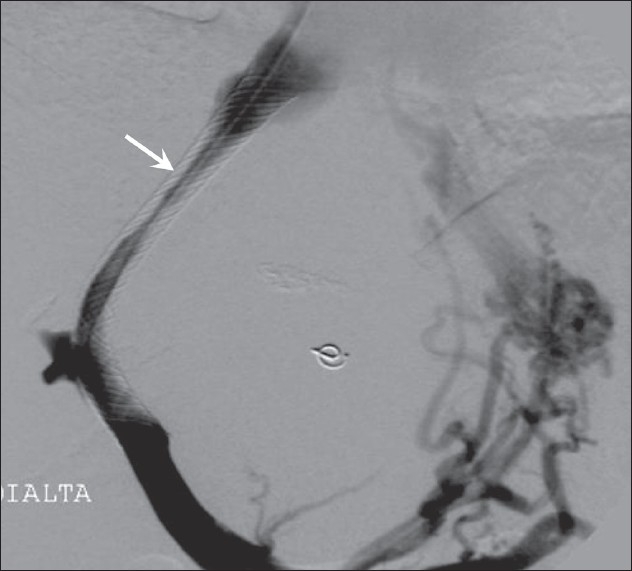
Mid-shunt stenosis (arrow) in a Wallstent, causing reappearance of varices and ascites. This was treated by balloon dilatation

**Figure 3:**
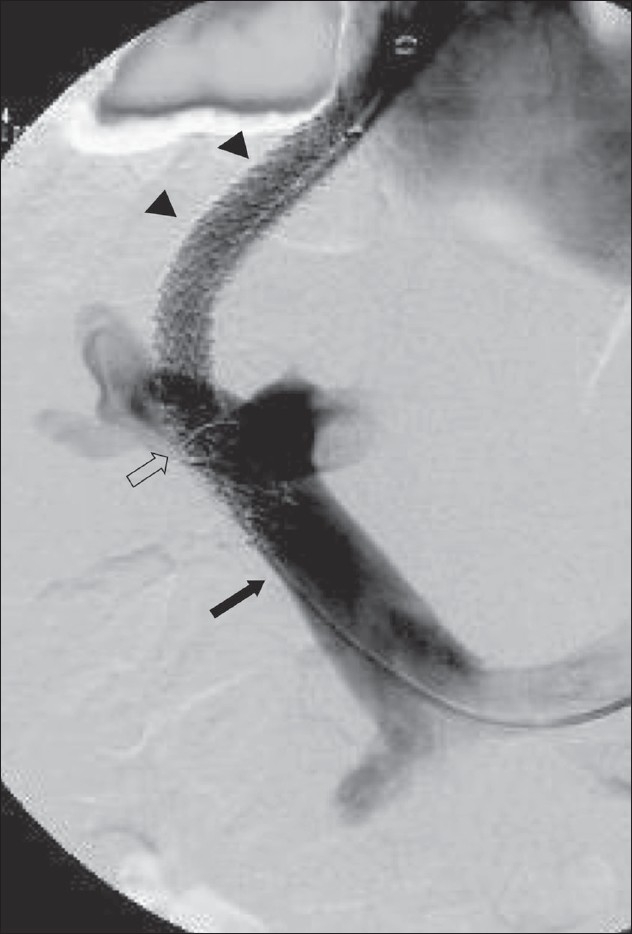
The Viatorr stent-graft device has a bare lower end that lies in the portal vein (arrow) and an upper covered end that bridges the liver parenchymal tract (arrowheads); the transition between the two is marked by an opaque ring (open arrow). When appropriately positioned, the bare end in the portal vein allows flow into the intrahepatic portal veins and the covered segment prevents intimal hyperplasia from blocking the shunt

The long-term results of TIPS are impaired by the high rate of shunt dysfunction from intimal hyperplasia and the resulting recurrence of symptoms [Figure 2]. The primary patency rate at 1, 2, 3, 4, and 5 years is 25-66%, 5-42%, 21%, 13%, and 13%, respectively. If a regular Doppler or angiographic surveillance of TIPS is done, early shunt stenosis can be detected and patency enhanced by a secondary balloon dilation or restenting. The resulting primary assisted patency rates approximate 85%, 61%, 46%, 42%, and 36% at 1, 2, 3, 4 and 5 years, respectively, and the cumulative secondary patency rates increase to 85-96% and 64-90% at 1 and 2 years, respectively.[22] However, this calls for reliable follow-up, more interventions, and a cumulative increase in expenditure incurred. The introduction of stent-grafts has largely mitigated this problem, with 1-year primary patency rates of >80%[23,24] [Figure 3]. In studies comparing bare stents and stent-grafts, the latter have been shown to provide much better patency rates.[25]

Like any other portosystemic shunt, TIPS can be responsible for encephalopathy in up to 50% of patients. Fortunately, most of these episodes are mild and only medical management is necessary. In 3-7% cases, this encephalopathy can be severe or recurrent, necessitating a shunt reduction.[26]

## Recanalization of Hepatic Venous Outflow

**Figure 4 (A, B):**
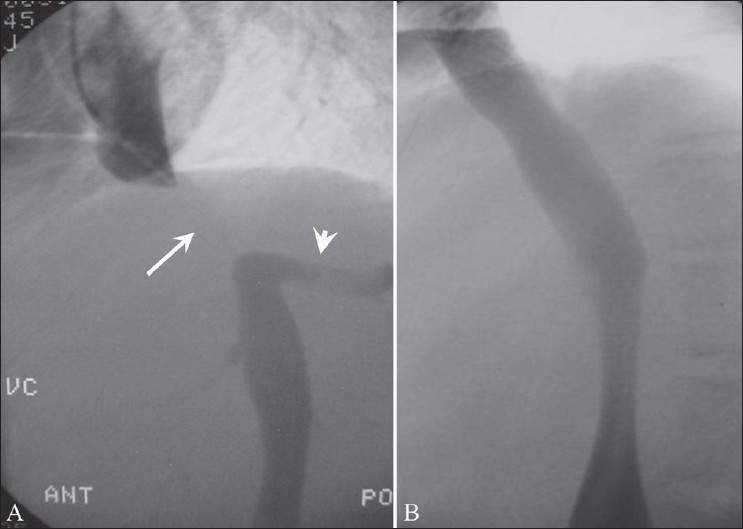
Budd-Chiari syndrome due to membranous obstruction of the inferior vena cava in the suprahepatic segment. Cavogram (A) shows a 4-cm long occlusion (arrow) of the inferior vena cava above the right hepatic vein (arrowhead); after needle fenestration of the occluded inferior vena cava through the femoral route, a stent is deployed across the occluded segment (B). The inferior vena cava is widely patent after stenting

**Figure 5 (A, B):**
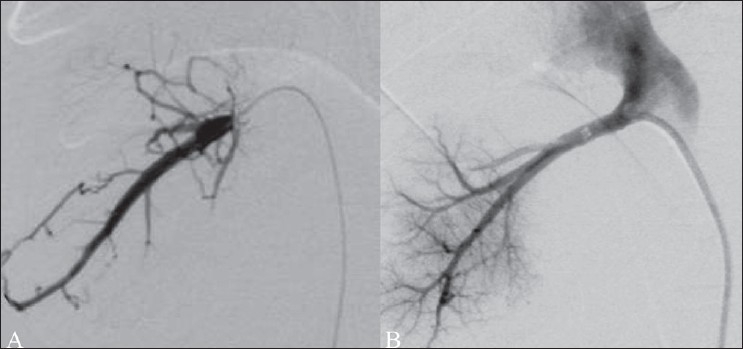
Budd-Chiari syndrome secondary to hepatic vein occlusion. The middle hepatic vein is obstructed close to its insertion into the inferior vena cava (A); after insertion of a balloon-expandable stent at the level of the occlusion, the hepatic venous outfl ow is restored (B)

**Figure 6 (A, B):**
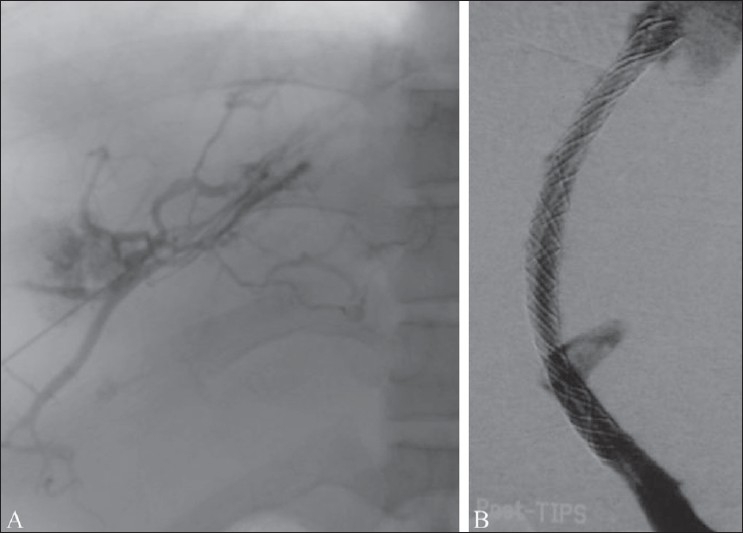
Budd-Chiari syndrome secondary to diffuse hepatic vein occlusion, not amenable to angioplasty. The classical ‘spider-web’ appearance of small intrahepatic venous collaterals is seen with diffuse thrombosis of the hepatic veins (A); transcaval TIPS directly connecting the inferior vena cava and the portal vein, effectively decompresses the portal venous system (B)

**Figure 7 (A, B):**
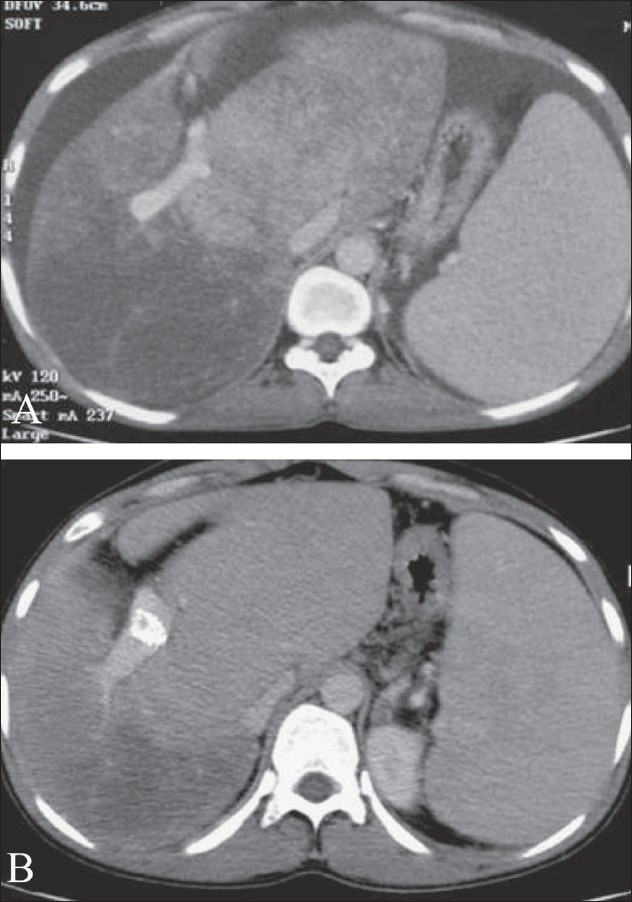
Contrast-enhanced CT scan of a patient with Budd-Chiari syndrome, before TIPS (A) and after TIPS (B), showing improvement in enhancement pattern of the liver parenchyma and regression of ascites following TIPS

Budd-Chiari syndrome includes all obstructions to the hepatic vein outflow at the level of the hepatic vein and/or the inferior vena cava. This causes hepatic congestion which, when left untreated, progresses to hepatic necrosis and fibrosis. The aim of treatment is to restore physiological flow, i.e., to recanalize the hepatic vein and/or the inferior vena cava by balloon angioplasty and stenting thus relieving the hepatic congestion and preventing progression to irreversible liver damage.[27] This is feasible if the obstruction is over a short segment [Figures 4 and 5]. Long segment hepatic vein occlusion is difficult to reopen and even if restored the reocclusion rates are extremely high. This subgroup of patients would need a portosystemic shunt. Surgical portocaval shunts are difficult to create, due to the large caudate lobe not allowing easy access to the portal vein. Also, portocaval shunts may not be successful, as the shunt often opens into a hypertensive cava due to caval compression by an enlarged caudate lobe. TIPS has increasingly been performed in such patients as it is associated with much less morbidity and can provide very gratifying results [Figures 6 and 7]. In addition, TIPS opens high into the inferior vena cava and is not affected by any compression by the enlarged caudate lobe. In the few published case series, ascites control is close to 100%, and there is improvement in liver function, obviating the need for transplantation in most cases.[28,29]

## Recanalization of the Portal Vein and Its Tributaries

**Figure 8 (A, B):**
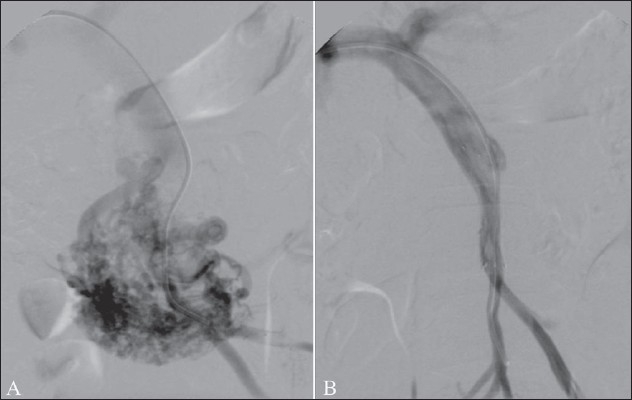
Large duodenal varices due to focal occlusion of the superior mesenteric vein (SMV), causing recurrent malena. After percutaneous transhepatic access to the portal vein (A), a catheter is advanced into the SMV. The venogram shows occlusion of the SMV, with filling of the duodenal variceal collaterals; the occluded segment is dilated and a stent deployed, following which there is direct flow from the SMV into the portal vein and no filling of the duodenal varices (B)

Extrahepatic obstruction of the portal vein or its branches can induce a focal PHT; this accounts for 5-10% of all cases of PHT. The cause of obstruction can be benign or malignant, and patients usually present with variceal bleeding, ascites, or abdominal pain. Recanalization of the blocked vein by angioplasty and stenting will reduce these symptoms and can be done either via a transjugular or a percutaneous transhepatic route [Figure 8].[30,31]

## Embolization of Arterioportal Fistulae

Arterioportal fistulae (APF) are a rare cause of PHT. They may be congenital or secondary to trauma, surgery, percutaneous biopsy or other liver procedures, and liver tumors. Although silent most of the times, some patients with large APFs can present with features of PHT such as bleeding, ascites, and splenomegaly. The preferred treatment is transarterial embolization of the feeding artery, using coils, detachable balloons, or glue. The procedure is usually successful, provided the fistula is not too large and is accessible.[32]

## Partial Splenic Embolization (PSE)

PSE is performed to diminish inflow of blood into the portal vein, with secondary reduction of the portal venous pressure. The procedure involves superselective catheterization and embolization of the intrasplenic arterial branches, usually with polyvinyl alcohol particles. This achieves reduction of portal vein pressure, reduction in splenic size, and improvement in hypersplenism-induced thrombocytopenia.[33] The results of treatment are good and the rate of serious complications (e.g., splenic abscess or sepsis) with current techniques is low.

## Percutaneous Transhepatic Variceal Embolization (PTE)

**Figure 9 (A, B):**
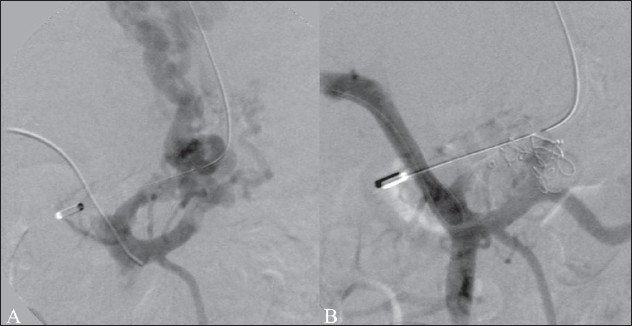
Percutaneous transhepatic embolization of varices in a patient with massive variceal bleeding. After percutaneous access into the portal vein (A), the venogram shows retrograde flow in the portal vein, filling varices from the left and posterior gastric veins; the gastric vein was selectively cannulated and embolized with coils (B). After this, there was reversal of flow in the portal vein and occlusion of the varices, providing short-term control of bleeding

PTE was the earliest intervention performed for portal hypertension and was first described by Lunderquist and Vang in 1974 to treat intractable variceal bleeding. In this technique, the portal vein is catheterized by a percutaneous transhepatic approach and the gastric vein feeding the varix is embolized with ethanol, steel coils, or cyanoacrylate glue [Figure 9]. When first described, PTE appeared to be a highly effective procedure, successfully controlling bleeding in 70-90% of patients. However, the underlying PHT was unaffected and, consequently, bleeding recurred in 38-70% of patients within 6 months and in 71-90% after 2 years. In addition, it carried a failure rate of 9%, particularly in patients with portal vein thrombosis or small livers with marked ascites.[34] PTE itself was responsible for inducing portal vein thrombosis in up to 36% of patients.[35] All these factors, and the emergence of endoscopic therapy (EST), led to a decline in the procedure; EST had better survival rates and lower rebleeding rates. The introduction of TIPS and BRTO further antiquated the procedure, and PTE is now very rarely performed.

## Balloon-occluded Retrograde Transvenous Obliteration of Varices (BRTO)

BRTO is a technique that is popular in Japan for control of gastric varices through a natural gastrorenal shunt. The technique involves advancing a balloon catheter from the femoral vein into the outlet of the gastrorenal shunt. Following balloon occlusion of the shunt, sclerosant (5% ethanolamine oleate) is injected retrogradely to fill the gastric varices. After adequate contact of the sclerosant with the variceal wall, the sclerosant is aspirated and the balloon catheter withdrawn. Considered by many to be as effective as TIPS in controlling gastric variceal bleeding, it has an added advantage in that it augments portal blood flow by occluding the natural shunt that takes blood away from the portal vein. This improves liver function in cirrhotic patients and also prevents encephalopathy, a problem commonly associated with TIPS.[36] However, occlusion of the gastrorenal shunt may aggravate existing esophageal varices or lead to the development of new ones, and this is one the most significant complications of BRTO.[37]

## Percutaneous Peritoneovenous Shunt

Surgical peritoneovenous shunts have been replaced by TIPS in most centers but are still offered to patients who cannot tolerate a TIPS. However, surgery in this high-risk group is associated with the morbidity of general anesthesia. These shunts can now be inserted by radiologists with less risk as the procedure is done under local anesthesia. In addition, venous entry puncture can be more precise, and access is easier, with USG guidance.[38]

## Conclusion

There are various interventional procedures that can be offered to patients with PHT. The choice of the procedure is based on the etiology of PHT, the symptoms, the clinical status, and the results of imaging studies. Most procedures now offer high success rates, good mid- and long-term results, and significantly less morbidity than the corresponding surgical procedures, and this has led to the emergence of interventional radiology as the procedure of choice in controlling PHT and its complications.
